# Group Training of Stress Management vs. Group Cognitive-Behavioral Therapy in Reducing Depression, Anxiety and Perceived Stress Among HIV-Positive Men

**Published:** 2013

**Authors:** Akbar Hemmati Sabet, Javad Khalatbari, Maryam Abbas Ghorbani, Mohammad Haghighi, Mohammad Ahmadpanah

**Affiliations:** 1 Islamic Azad University, Tonekabun Branch, Iran**.**; 2 Behavioral Disorders and Substances Abuse Research Center, Hamadan University of Medical Science, Hamadan, Iran.

**Keywords:** Anxiety, Cognitive-Behavioral Therapy (CBT), Depression, Group Training of Stress Management, HIV

## Abstract

** Objective: **To compare the effectiveness of group training of stress management with cognitive-behavioral therapy (CBT) in reducing depression, anxiety and stress perceived among HIV-positive men.

**Methods:**Inthis semi-experimental study, three groups of HIV-positive men (CBT group, stress management group, and control group) including 15 patients in each group were compared regarding depression, anxiety, and stress using pre-test and post-test tools.

** Results: **Both interventions (CBT and stress management) were effective in reducing depression, anxiety and perceived stress. Evaluating adjustedmean showed the more effectivenessofthe group stressmanagementtraining than CBT.

**Conclusion: **Group stress management training is more effective than group CBT in HIV-positive mentodecreasedepression, anxiety and stress management.

**Declaration of interest:** None.

**Clinical Trial Registratio**n: URL: http://.irct.ir. Unique identifier: 2012121711782N1

## Introduction

The basic feature of HIV infection is the gradual demolition of physical ability as well as neurological-emotional phenomena (1). HIV-positive people have to fight with fear associated with diseases such as disability, increasing independence, reducing control of the body and pain. Nature of stressful agents is one of the most fundamental aspects of the people with HIV infection and AIDS. Although not unexpected, a range of psychological disturbances from a relatively light state, such as sick like apathy, feeling guilty, helplessness and frustration to extreme conditions such as anxiety, depression and sometimes suicidal thought disorders have been seen in these patients(2).

Anxiety is an unpleasant and inappropriate feeling, whichis usually associated with physiological symptoms. Depression or mixed anxiety and depression were identified in 15.5% of outpatients at a dedicated Tanzanian HIV/AIDS care center, with 4.5% suffering from other anxiety disorders (3).

Several techniques are used in the group psychotherapy for which cognitive therapies one of the most common methods of treatment of depression, anxiety and perceived stress in HIV-positive men. A key strategy for effective treatment is offering group therapy. Perhaps the first person who seriously paid attention to this work was a psychiatrist, Maxwell Jones. He realized the lack of adequate resources to treat veterans suffering from war neurosis during World War II, and found that these people can be treated quite effectively in treatment groups. Parallel to the emergence of new treatments, the idea of treating people in groups still remained. Groups were formed for more humanistic treatments; Gestalt therapy and interactive analysis. With the formation of behavioral therapy in the early 1960's, successful efforts began to use desensitization techniques in the groups.

The same happened for cognitive therapy. Two milestones in the development of behavioral therapy were a published in a studyin1977 and a treatment guide.Since then, cognitive-behavioral therapy (CBT) has become the dominant psychotherapy in most Western countries, and is used as a frame work in many empirically validated treatments (4). Immunization against stress is also one aspect of life style and some studies have confirmed the relationship between the physical and health factors.In other word, stress develops in the relationship between the individual and the environment in which the individuals assess relationship very stressful and found he is unable to control himself. The importance of this issue is clear when we consider the important role of emotional factors in preventing the rapid development of physical illness such as cancer, stroke, heartconditions, and psychosomatic disease (5).

According to a study regarding group CBT in improving adherence to medication and depression among HIV- positive patients, the subjects showed significant improvement in adherence to medication and reduced depression within three months. The authors concluded that CBT is a potentially effective method to combat depression and poor adherence to medication for people with HIV (6). In another study, the efficiency of the 10-week interventions of group cognitive-behavioral stress management on 210HIV-positive heterosexual and homosexual men was examined. Group cognitive-behavioral stress management interventions reduced stress and depression, increased psychological adjustment, increased coping skills, increased social support and improved quality of life (7). In another study, stress management training by cognitive-behavior technique and among women in the early stages of breast cancer showed that women who were tested in the stress management training had positive feelings in response to their breast cancer tests which simultaneously caused the late improvement of immunity and cellular immunity (8).

Stress management training by cognitive-behavioral technique has been shown to be effective on depression and blood sugar control in type 2 diabetic patients(9). In conducted a research as “The effects of group cognitive therapy in reducing depression in male patients withHIV-positive” in two experimental groups and test groups completed 10 sessions of cognitive therapy. Results showed that group cognitive therapy significantly was effective in reducing depression in HIV-infected patients (10). In a 5-years study ,The results showed that cognitive-behavioral group interventions is effective to reduce social isolation, cause reduction in negativethoughts and depression, reducing the destruction of cells CD4 + T, improve adaptive coping methods, increase healthy behaviors, treatment compliance, decrease drug abuse, reduce inappropriate sexual behavior, reduced is ease development and improve quality of life in HIV-positive individuals in the experimental group in compared with the control group(11).

In another research, the result showed that group training of stress management and cognitive group therapy are both effective in reducing student social phobia (12).

The authors intended to determine whether group training of stress management by CBT and group cognitive therapy techniques for depression, anxiety and stress in HIV-infected men is effective or not. This research can help mental health professionals to treat HIV-infected patients by focusing on recognition and specify the path and not scattered mental disorders such as depression, anxiety and stress and also help them in order to predict the future behavior of patients in the treatment process and apply effective treatments for helping those with mental problems.

## Materials and Methods

In thissemi-experimental research, three groups (two experimental groups and one control group) were studied using pre-test and post-test. The study population included all HIV-positive male patients identified by health consultant clinicsof Mazandaran province, Iran in 2010 and 2011. Due to thelimited number of female patients, they were not included in the study. HIV-positive patients were assessed in terms of depression, anxiety and stress (13). The final sample was selected randomly by file number and placed in 3 groups of 15 people each, including two experimental groups (i.e., CBT and training stress management) and one controlgroup. Patients diagnosed with depression, anxiety and perceived stress were those who scored 16 or more intheir depression scores, 16 or more in their anxiety scores and 24 or more in their stress scores in the questionnaires of depression, anxiety, and stress scale (13).

The important application of the scale is to measure the severity of major symptoms of depression, anxiety and perceived stress. The patients were informed about the confidentiality of the data gathered and were informed that codes would be used instead of their names. Moreover, the study protocol received ethics committee confirmation from Azad University of Ramsar, Iran. 

Time for each treatment session (CBT) was 1.5 hours, and this treatment procedure was performed in 10 sessions. Time for each stress management training session was 1.5 hours and this procedure was run in 10 sessions. The control group received no treatment.At the end of treatment, scales of depression, anxiety and stress for all three groups were measured again and the impact of stress management training and group CBT on reducing depression, anxiety and stress was studied. For descriptive analysis of data, statistical indicators like mean and standard deviation (±SD) were used and in deductive statistic, covariance analysiswas used. All analyses were done using the SPSS software for Windows (ver. 18.0).

## Results

Table 1 shows pre-test and post-test scores of depression, anxiety and perceived stress in experimental and control groups.

Table 2 presents the results of covariance analysis of depression, anxiety and perceived stress.

In all groups the alpha level adjusted by Ben Fruny (0.003) was used.

After adjusting mean pretest levels of depression (27.91), anxiety (22.67), and perceived stress (30.80) and calculated F, it can be concluded that the differences between the post-test scores of these variables in both experimental groups and control group are significant. 

After the significance of calculated F using hoc tests was realized, comparative evaluation of effectiveness of CBT and stress management training on the rate of depression, anxiety and perceived stress were performed (Table 3). Both interventions (CBT and stress management) were effective in reducing depression, anxiety and perceived stress. Evaluating adjusted mean showed the more effectivenessofthe group stressmanagementtraining than CBT.

**Table 1:** Average and standard deviation of pre-test and post-test scores in experimental and control groups

**Group**	**Cognitive-behavioral Training**		**Training stress management**		**Control group**	
	**Mean**	**SD**	**Mean**	**SD**	**Mean**	**SD**
**Pre-test depression**	29.07	6.181		27.20	6.038	27.47
**Post-test depression**	23.73	4.949		21.47	4.926	28
**Pre-test anxiety**	24	6.761		21.87	6.523	22.13
**Post-test anxiety**	17.33	5.589		15.73	5.650	22.53
**Pre-test Perceived stress**	31.47	4.438		29.47	4.438	31.47
**Post-test Perceived stress**	24.40	4.014		22.27	4.061	32.53

**Table 2:** Results of covariance analysis of depression, anxiety and perceived stress

	**Diffraction source**	**Sum square** ** SS**	**degrees of freedom f** **δ**	**Mean square** **ms**	**F**	**P**	**Effect size Eta**	**Test power**
**group**	**Depression**	345.218	2	177.109	19.532	0.001	0.500	1.000
	**Anxiety**	425.861	2	212.931	20.267	0.001	0.510	1.000
	**Stress**	684.995	2	342.498	55.013	0.001	0.739	1.000

**Table 3:** Analysis of covariance between therapies of depression, anxiety, and perceived stress based on cognitive-behavioral and stress management training

	**Diffraction source**	**Sum square**	**Degrees of freedom **	**Mean square**	**f**	**P**	**Mean difference**
**group**	**Depression**	232.469	1	232.469	25.638	0.001	5.650
	**Anxiety**	324.144	1	324.144	30.852	0.001	6.672
	**Perceived stress**	474.666	1	474.666	76.242	0.001	8.073

## Discussion

The effectiveness of stress management training by cognitive behavioral technique and group cognitive therapy in reducing depression, anxiety and perceived stress in HIV positive men is different. The data from this study showed that, there is significant difference between the two groups of group training of stress management and group cognitive therapy on depression, anxiety and perceived stress in HIV-positive men which this difference is in favor of group training of stress management by cognitive-behavior technique than group cognitive therapy. 

We did not find any article published in the literature regarding evaluation of group training in stress management and group cognitive therapy on reducing depression, anxiety and perceived stress in HIV-positive men. But in other studies impact of stress management training and group CBT have been assessed separately on depression, anxiety and perceived stress in HIV-positive men which all are aligned and parallel with the results of this study. It can be stated that, cognitive behavioral stress management success is due to the CBT group. Skills trained to reduce anxiety, muscle relaxation techniques, which include gradual, guided imagery, autogenic, meditation, diaphragmatic breathing are all discussed. 

The limitations of this study are the inability to control confounding variables. These variables are personality variables, physical and mental (memory, talent, desire and motivation) and affective variables (mood, emotions, etc.) as well as economic conditions, social and cultural subjects.

## Conclusion

The results indicate that the two methods of group stress management training and group CBT are effective in reducing depression, anxiety and perceived stress in HIV-positive men. Group stress management training is more effective than CBT. 

## Authors’ Contributions

AHS and JKh conceived and designed the evaluation. MAGh collected the clinical data. MH interpreted the clinical data. MA performed the statistical analysis and drafted the manuscript. All authors read and approve the final manuscript.
